# Analysis of the detection rate and related factors of fatty liver disease in physical examination of healthy population in Chengdu district

**DOI:** 10.1097/MD.0000000000035087

**Published:** 2023-09-08

**Authors:** Qian Han, Jiaojiao Guo, Ling Gong, Changqing Liu, Fan Zhang

**Affiliations:** a Department of Health Management Center, General Practice Medical Center, West China Hospital, Sichuan University, Chengdu, Sichuan, China; b Department of Operating Room of West China Hospital, West China School of Nursing, Sichuan University, Nursing Key Laboratory of Sichuan, Chengdu, Sichuan, China.

**Keywords:** Chengdu region, fatty liver, health examination, influence factor

## Abstract

**Background:**

In the present study, we analyzed the detection rate and related influencing factors of fatty liver in the health examination population in Chengdu area.

**Methods:**

The case-control study was performed to compare the gender, age (years), body mass index (BMI), smoking, drinking, abnormal lipid metabolism, hypertension, hyperglycemia, hyperuricemia Is there any statistically significant difference in the detection rate of diseases such as metabolic syndrome, and logistic regression analysis is conducted to analyze the comprehensive impact of each influencing factor on the prevention of fatty liver disease.

**Results:**

Among 14,426 survey subjects, a total of 6717 patients with fatty liver were detected, with a detection rate of 47.22%. There are significant differences in the incidence of fatty liver disease among different gender groups, with the incidence rate in males being significantly higher than that in females (*P* < .05); The incidence of fatty liver in elderly subjects was significantly higher than that in middle-aged and young subjects (*P* < .05); The prevalence rate of individuals with a BMI > 24 was significantly higher than that of individuals with a BMI < 24 (*P* < .05). The prevalence of fatty liver in the population with abnormal lipid metabolism, hypertension, hyperglycemia, hyperuricemia, metabolic syndrome and other diseases was significantly higher (*P* < .05); After stratified analysis by gender and age, the incidence of fatty liver in males was significantly higher than that in females in the 3 age groups < 60 years old (*P* < .05); In the age group ≥ 60 years old, the difference in the incidence of fatty liver disease between males and females was significantly reduced, and the difference was not statistically significant (*P* > .05).

**Conclusion:**

The health screening of patients with fatty liver should be carried out regularly, and attention should be paid to the intervention and prevention of overweight people and people with basal metabolism diseases such as hyperglycemia and hypertension, so as to reduce the incidence of fatty liver.

## 1. Introduction

Fatty liver disease (FLD) can lead to liver fibrosis, cirrhosis, and even liver cancer, and is one of the serious diseases that seriously threaten the life and health safety of people in China.^[1,2]^ FLD refers to the excessive accumulation of fat in the liver, which makes the steatosis of the liver reach more than 33.3% per unit of histological area or more than 5% of the wet weight of the liver.^[3,4]^ In recent years, the incidence of FLD in China has been increasing year by year, and it has become the second largest liver disease after hepatitis, with a 10% chance of developing into cirrhosis, posing a serious threat to human health.^[5,6]^ FLD can be divided into alcoholic fatty liver and nonalcoholic fatty liver.^[7]^ According to relevant studies, FLD is closely related to heredity and living environment, and is closely related to overweight, type 2 diabetes, metabolic syndrome (MetS) and arteriosclerotic cardiovascular disease.^[8–10]^ In order to explore the detection rate and related influencing factors of fatty liver in the health examination population in Chengdu, our research group conducted a case-control study on 14,226 subjects and 6717 patients with fatty liver who underwent physical examination at the Examination Center of West China Hospital, Sichuan University. The results are reported as follows.

## 2. Object and method

### 2.1. Research subjects

From January 2019 to December 2019, a survey was conducted on 14,226 subjects who came to the physical examination center of West China Hospital of Sichuan University for physical examination. It was gotten ethics approval from the ethics committee of General Practice Medical Center, West China Hospital, Sichuan University. Among them, 6717 patients with fatty liver were diagnosed. Diagnostic criteria: the imaging diagnostic criteria of fatty liver specified by the Fatty Liver and Alcoholic Liver Disease Group of the Liver Credit Society of the Chinese Medical Association in 2002 were used: the liver parenchyma was dotted hyperechoic; Deep liver echo attenuation; The display of intrahepatic blood vessels is unclear. If the above 3 criteria meet the first criterion and any one of the second or third criteria is added, it can be diagnosed as fatty liver.^[11]^ Inclusion criteria: between the ages of 20 and 80; No severe organ dysfunction; Patients with concomitant tumors, hematological systems, immune systems, and mental disorders. Exclusion criteria: estimated survival time < 1 year; Those who do not agree with the requirements of this trial, or cannot cooperate with treatment, or cannot follow up on time. Exclusion criteria: respondents do not cooperate; The attitude of the survey respondents in answering questions is not serious.

## 3. Grouping and investigation content

Fourteen thousand two hundred twenty-six study subjects were divided into a case group of 6717 patients who were diagnosed with fatty liver. The remaining subjects who were not diagnosed with fatty liver were divided into a control group, and a case-control study was conducted on the 2 groups of study subjects. The investigation contents include: gender, age (years), body mass index (BMI), drinking, smoking, whether there is abnormal lipid metabolism, hypertension, hyperglycemia, hyperuricemia, MetS and other diseases.

## 4. Investigation content diagnostic criteria

1. Diagnosis of abnormal lipid metabolism is based on 1 or more of the following conditions: Total serum cholesterol > 200 mg/dL or low-density lipoprotein cholesterol > 120 mg/dL; Serum triglycerides > 150 mg/dL; High-density lipoprotein cholesterol (HDL-C) < 35 mg/dL.^[12,13]^
2. According to the 2010 Guidelines for Diagnosis and Treatment of Primary Gout of the Chinese Society of Rheumatology, men with uric acid concentration > 7 mg/dL and women with uric acid concentration > 6 mg/dL are defined as hyperuricemia. According to the diagnostic criteria recommended by diabetes Branch of Chinese Medical Association: overweight or obesity (BMI ≥ 25).
3. The criteria for determining the components of MetS issued by the Joint Committee for the Development of Guidelines for the Prevention and Treatment of Adult Lipids in China in 2016^[14,15]^: Abdominal obesity: waist circumference > 90 cm for males and > 85 cm for females; The level of triglycerides (TG) in blood increases, with TG ≥ 1.70 mmol/L (150mg/dL); Blood HDL-C decreased, HDL-C < 1.0 mmoL/L (40 mg/dL); Hypertension: Individuals who have been diagnosed with hypertension and treated, or (and) systolic blood pressure ≥ 130 mmHg and (or) diastolic blood pressure ≥ 85 mmHg; Fasting plasma glucose (GLU) ≥ 6.1 mmoL/L (110 mg/dL) or blood GLU ≥ 7.8 mmoL/L (140mg/dL) 2 hours after GLU load or a history of diabetes. A score of ≥ 3 items in the MetS group can be diagnosed as MetS. Fasting blood GLU ≥ 6.1 mmol/L and those who have been diagnosed with diabetes.

## 5. Quality control

After the questionnaire design is completed, select medical staff who are familiar with the local situation as investigators and provide them with strict and unified training. Each item in the questionnaire is required to be surveyed and filled out strictly according to the coding instructions. At the same time, it is required to complete a preliminary review of the form at the investigation site, mainly checking whether the basic information is complete, supplementing any unnecessary blank or missing items, and conducting a preliminary inspection for obvious filling errors or logical errors.

## 6. Statistical analysis

Statistical analysis was conducted using SAS9.4 software (https://support.sas.com/en/documentation/install-center.html). Two sets of measurement data are represented by “x ± s” and compared using 2 independent sample *t* tests; Two sets of classification data were compared using a 4 grid X2 test; The prevalence of fatty liver disease in different age groups and BMI levels was compared using linear trend X2 test; The multivariate analysis was conducted using binary logistic regression analysis, and the variable assignments are shown in Table 1. Inspection level α = 0.05.

**Table 1:** Assignment of factors influencing fatty liver.

Variable	Assignment
Dependent variable	
Occurrence of FLD	0 = Normal;1 = FLD
Independent variables	
Agent	0 = Female;1 = Male
Age	0=“<39”1; =“40~”; 2=“50~”; 3=“60~”; 4=“≥70”
BMI	0=“<24”; 1=“24~28”; 2=“>28”
Drinking	0 = Yes; 1 = No
Abnormal lipid metabolism	0 = Yes; 1 = No
Hypertension	0 = Yes; 1 = No
Hyperglycemia	0 = Yes; 1 = No
Hyperuricemia	0 = Yes; 1 = No
Metabolic syndrome	0 = Yes; 1 = No

BMI = body mass index, FLD = fatty liver disease.

## 7. Result

### 7.1. Basic information of survey subjects and prevalence of fatty liver disease

Among 14,226 survey subjects, there were significant differences in the prevalence of fatty liver disease among different gender groups, with male prevalence significantly higher than female (*P* < .001); The incidence of fatty liver in elderly subjects was significantly higher than that in middle-aged and young subjects, with a statistically significant difference (*P* < .001); At the same time, the prevalence rate of the population with a BMI > 24 was significantly higher than that of the population with a BMI < 24, and the difference was statistically significant (*P* < .001). The prevalence of fatty liver in the population with smoking, drinking, abnormal lipid metabolism, hypertension, hyperglycemia, hyperuricemia, MetS, and other diseases was significantly higher (*P* < .001), as shown in Table 2. Through comparison of various inspection indicators, BMI, systolic pressure, diastolic pressure, GLU, uric acid, TG, low-density lipoprotein, waist circumference, liver fibrosis measured value CAP, liver fibrosis measured value E of patients with fatty liver are higher than those of healthy control people (*P* < .001), while high-density lipoprotein is lower than those of healthy control people (*P* < .001), as shown in Table 3.

**Table 2:** Basic information and fatty liver disease status of the surveyed physical examination personnel.

Factors	Number of people undergoing physical examination	FLD	*X^2^* value	*P* value
Male	8222 (57.8)	4844 (58.92)	1069.8085	<.001
Female	6004 (42.2)	1873 (31.2)		
Age (yr old)				
≥70	371 (2.61)	186 (50.13)	141.2114212	<.001
60~	1733 (12.18)	926 (53.43)		
50~	4172 (29.33)	2148 (51.49)		
40~	4983 (35.03)	2358 (47.32)		
<39	2967 (20.86)	1099 (37.04)		
BMI				
<18	323 (2.27)	47 (14.55)	2698.738	<.001
18–24	685 (48.19)	1769 (25.8)		
>24	7047 (49.54	4901 (69.55)		
Smoking				
Yes	4746 (33.36)	2727 (57.46)	299.7964	<.001
No	9480 (66.64)	3990 (42.09)		
Drinking				
Yes	717 (50.44)	3991 (55.62)	409.9201	<.001
No	705 (49.56)	2726 (38.67)		
Hypertension				
Yes	1381 (97.09)	6442 (46.64)	63.1296	<0.001
No	414 (2.91)	275 (66.43)		
Hyperglycemia				
Yes	1706 (11.99)	1177 (68.99)	368.807	<.001
No	1252 (88.01)	5540 (44.25)		
Hyperuricemia				
Yes	3400 (23.9)	2281 (67.09)	707.9122	<.001
No	10,826(76.1)	4436 (40.98)		
Abnormal lipid metabolism				
Abnormal	8582 (60.33)	5033 (58.65)	1133.8549	<.001
Normal	5644 (39.67)	1684 (29.84)		
Metabolic syndrome				
Yes	3390 (23.83)	2681 (79.09)	1813.6917	<.001
No	1083 (76.17)	4036 (37.25)		
Total	14,226	6717		

BMI = body mass index, FLD = fatty liver disease.

**Table 3:** Comparison of various examination indicators between non FLD patients and healthy people($\bar{x}\pm s$).

Variable	Healthy people (n = 7505)	FLD (n = 6703)	t value	*P* value
BMI (kg/m^2^)	22.55 ± 2.70	25.80 ± 2.94	−68.37	<.001
Systolic pressure (mm Hg)	115.4 ± 15.90	123.4 ± 15.85	−29.7	<.001
Diastolic pressure (mm Hg)	71.00 ± 10.30	76.35 ± 10.71	−30.3	<.001
GLU (mmol/L)	5.01 ± 1.04	5.51 ± 1.56	−22.94	<.001
UA (mmol/L)	316.3 ± 82.64	374.9 ± 87.21	−41.11	<.001
TG (mmol/L)	1.32 ± 0.86	2.20 ± 1.73	−39.08	<.001
LDL (mmol/L)	2.88 ± 0.80	3.07 ± 0.83	−13.94	<.001
HDL (mmol/L)	1.54 ± 0.40	1.25 ± 0.34	45.77	<.001
Waist (cm)	77.30 ± 8.94	87.87 ± 8.93	−70.25	<.001
CAP (dB/m)	204.2 ± 26.67	280.2 ± 36.37	−143.26	<.001
E (kPa)	4.78 ± 9.15	5.02 ± 1.68	−2.22	.0266

BMI = body mass index, FLD = fatty liver disease, LDL = low-density lipoprotein, TG = triglyceride, UA = uric acid.

## 8. Comparison of detection rates of fatty liver disease among different ages and genders

Out of 14,226 study subjects, 6717 cases of fatty liver were detected, with an overall crude prevalence ate of 47.22%, with a prevalence rate of 58.92% for males and 31.2% for females. After stratified analysis by gender and age, the incidence of fatty liver disease in males was significantly higher than that in females in the 3 age groups < 60 years old, and the differences were statistically significant (*P* < .01); In the age group ≥ 60 years old, the difference in the incidence of fatty liver disease between males and females is significantly reduced. The incidence of fatty liver disease increases with age among different genders, as shown in Table 4.

**Table 4:** Logistic regression analysis results of influencing factors of fatty liver.

Variable	*SE*	*WaldX^2^* value	*freedom*	*P* value	*OR value*	*95% CI*
Gender	0.0555	22.6988	1	<.001	1.302	1.168–1.452
Age	0.00201	8.71	1	.0032	1.006	1.002-1.01
BMI	0.0088	1413.649	1	<.001	1.392	1.368–1.416
Smoking	0.025	0.9476	1	.3303	1.05	0.952–1.158
Drinking	0.0259	2.1167	1	.1457	0.927	0.838–1.027
Abnormal lipid metabolism	0.0443	158.7788	1	<.001	1.748	1.603–1.907
Hypertension	0.1233	8.5933	1	.0034	0.697	0.547–0.887
Hyperglycemia	0.071	21.3929	1	<.001	1.389	1.208–1.596
Hyperuricemia	0.0494	47.3183	1	<.001	1.405	1.275–1.548
Metabolic syndrome	0.0602	71.206	1	<.001	1.662	1.477–1.87

BMI = body mass index.

## 9. Logistic regression analysis results of influencing factors of fatty liver disease

Fitting the binary logistic regression equation showed statistical significance (*P* < .01). The final eight influencing factors were included in the regression equation, namely: gender (OR = 1.302), age (OR = 1.006), BMI (OR = 1.392), abnormal lipid metabolism (OR = 1.748), hypertension (OR = 0.697), hyperglycemia (OR = 1.389), hyperuricemia (OR = 1.405), and MetS (OR = 1.662). See Table 5.

**Table 5:** Logistic regression analysis results of influencing factors of fatty liver.

Variable	*SE*	*WaldX^2^* value	*freedom*	*P value*	*OR value*	*95% CI*
Gender	0.0555	22.6988	1	<.001	1.302	1.168–1.452
Age	0.00201	8.71	1	.0032	1.006	1.002-1.01
BMI	0.0088	1413.649	1	<.001	1.392	1.368–1.416
Smoking	0.025	0.9476	1	.3303	1.05	0.952–1.158
Drinking	0.0259	2.1167	1	.1457	0.927	0.838–1.027
Abnormal lipid metabolism	0.0443	158.7788	1	<.001	1.748	1.603–1.907
Hypertension	0.1233	8.5933	1	.0034	0.697	0.547–0.887
Hyperglycemia	0.071	21.3929	1	<.001	1.389	1.208–1.596
Hyperuricemia	0.0494	47.3183	1	<.001	1.405	1.275–1.548
Metabolic syndrome	0.0602	71.206	1	<.001	1.662	1.477–1.87

BMI = body mass index.

## 10. Discussion

With the continuous improvement of people’s living standards, there have been significant changes in their way of life, including travel and dietary habits, which invisibly leads to a continuous increase in the incidence of some habitual diseases. According to relevant reports, FLD is a kind of common lifestyle disease. Bad lifestyle, such as eating well, doing little exercise and overeating, has led to an increasing incidence rate of fatty liver.^[16]^ FLD can be divided into alcoholic fatty liver and nonalcoholic fatty liver, with 90% of cases attributed to metabolic factors, namely nonalcoholic fatty liver.^[17]^ According to relevant studies, the risk of fatty liver disease is much higher in people who have a long-term high salt, high oil, and high sugar diet than in those who have a normal diet.^[14]^ At the same time, relevant research data show that the liver function of patients with basic metabolic diseases is significantly lower than that of normal people, and the incidence rate of fatty liver is much higher than that of normal people.^[15,18]^ It can be seen that the occurrence of fatty liver is closely related to our own metabolism and daily diet.

This study found significant differences in the prevalence of fatty liver disease among different gender groups, with male prevalence significantly higher than female prevalence. Multivariate logistic regression analysis showed that the influence of gender was OR = 1.302, which may be related to male lifestyle habits. According to research data, the incidence of smoking and alcohol addiction in males in China is much higher than that in females. Due to their own dietary habits or work and social interactions, men have significantly higher levels of alcohol intake and high oil and salt intake than women, which can lead to a decrease in liver function levels in this group of men, leading to a higher incidence of fatty liver disease.^[19,20]^ At the same time, the research results show that the prevalence rate of fatty liver in elderly subjects is significantly higher than that in young and middle-aged subjects, and the influence of age is OR = 1.006, which may be because people’s basal metabolism rate decreases with age, their own metabolic capacity is poor, and their metabolic level is low. When they eat food with high oil and salt, it is more difficult to digest and absorb it. At the same time, with the accumulation of years, the level of liver function is also decreasing, The incidence of fatty liver disease in elderly people is higher than that in young people.^[21]^

This study found that the prevalence of fatty liver disease was significantly higher in the population with a BMI > 24 than in the population with a BMI < 24, and the impact of body weight on fatty liver disease was OR = 1.392; The relevant data shows that the risk of fatty liver disease in overweight and obese individuals is 1.799 times and 2.527 times higher than that in healthy individuals. This may be related to abnormal insulin metabolism, and insulin resistance is believed to be a bridge between overweight, obesity, and fatty liver, and they are mutually causal.^[22,23]^ The basis of IR is the increased secretion of hormones and cytokines from adipocytes caused by obesity, and related endocrine changes affect the storage and mobilization of fat, thereby altering insulin sensitivity.^[24]^

The study found that the effects of abnormal lipid metabolism, hypertension, hyperglycemia and hyperuricemia on fatty liver were OR = 1.748, OR = 0.697, OR = 1.389, and OR = 1.405, respectively. Abnormal lipid metabolism, hypertension, and hyperglycemia are often mutually causal factors that collectively affect people’s metabolism. Blood pressure, as a metabolic indicator, is often associated with multiple metabolic cardiovascular risk factors such as obesity, dyslipidemia, fatty liver, and is closely related to coronary heart disease, as well as GLU and lipid metabolism disorders. Abnormal lipid metabolism can lead to abnormal accumulation of fat in the body, and after passing through the body’s circulation, undigested fat in the body can be converted into carbohydrates, leading to an abnormal increase in blood sugar levels in the body.^[25]^ Hyperuricemia is a metabolic disease with elevated serum uric acid, which can not only cause gout, but also aggravate obesity. In recent years, it has an increasing and younger age trend, most of whom are asymptomatic. During physical examination, it was found that the level of uric acid was higher than normal.^[26]^ Previous studies have shown that the serum uric acid level of fatty liver patients is higher than that of nonfatty liver patients. The specific mechanism is that hyperuricemia causes insulin resistance, which leads to disorder of glycolysis and free fatty acid metabolism, and excessive fat is deposited in the liver, forming fatty liver.^[27]^

In the present study, the diagnostic criteria for fatty liver are based on imaging examination methods. Although this is the simplest, most effective, and most direct method, there are still some shortcomings. For example, it had better to combine biochemical tests and liver and spleen CT scans to further confirm the detection rate of fatty liver. In addition, the data related to live function tests such as ALT, ASTALP, GGT, albumin, bilirubin, and INR should be added, as well as the information related to CT scan of patients and NAFLD score should be added. It would be added in further examination for fatty liver detection. To sum up, the prevalence rate of fatty liver among health examination population in Chengdu is related to age, gender, hypertension, hyperglycemia, hyperuricemia, metabolic abnormalities, obesity and other risk factors. Targeted preventive measures should be taken to control it, so as to reduce the prevalence rate of fatty liver and improve people’s healthy living standards as a whole.

## Author contributions

**Conceptualization:** Fan Zhang.

**Data curation:** Qian Han, Changqing Liu, Fan Zhang.

**Formal analysis:** Jiaojiao Guo, Changqing Liu.

**Methodology:** Qian Han, Jiaojiao Guo.

**Project administration:** Ling Gong.

**Software:** Jiaojiao Guo, Ling Gong, Changqing Liu, Fan Zhang.

**Validation:** Qian Han, Jiaojiao Guo, Changqing Liu.

**Visualization:** Ling Gong.

**Writing – review & editing:** Ling Gong.

**Writing – original draft:** Changqing Liu.
